# Supplementary figures and images for: Alpha-Frequency Stimulation Enhances Synchronization of Alpha Oscillations with Default Mode Network Connectivity
Source: eNeuro. 2025 Mar 20;12(3):ENEURO.0449-24.2025. doi: 10.1523/ENEURO.0449-24.2025 (PMC11927933; doi:10.1523/ENEURO.0449-24.2025)

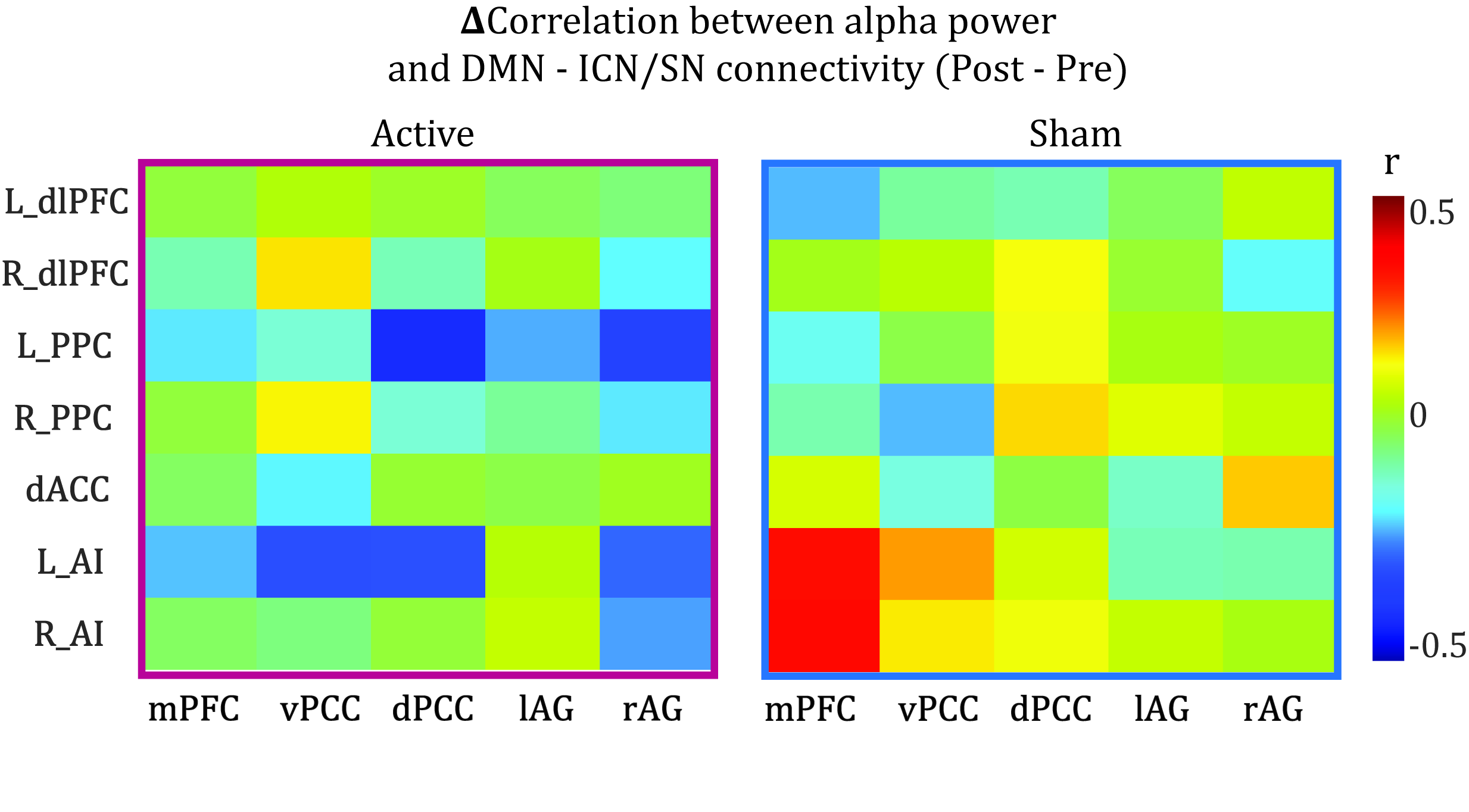

Supplement: Figure 2-1 — No effect of α-tACS on the dynamic coupling between alpha power and connectivity of DMN with the CEN and SN: Differential (Post – Pre) dynamic coupling matrices were comparable between Active and Sham groups. No significant effects of tACS were observed. Download Figure 2-1, TIF file. [file eneuro-12-ENEURO.0449-24.2025-s001.tif]

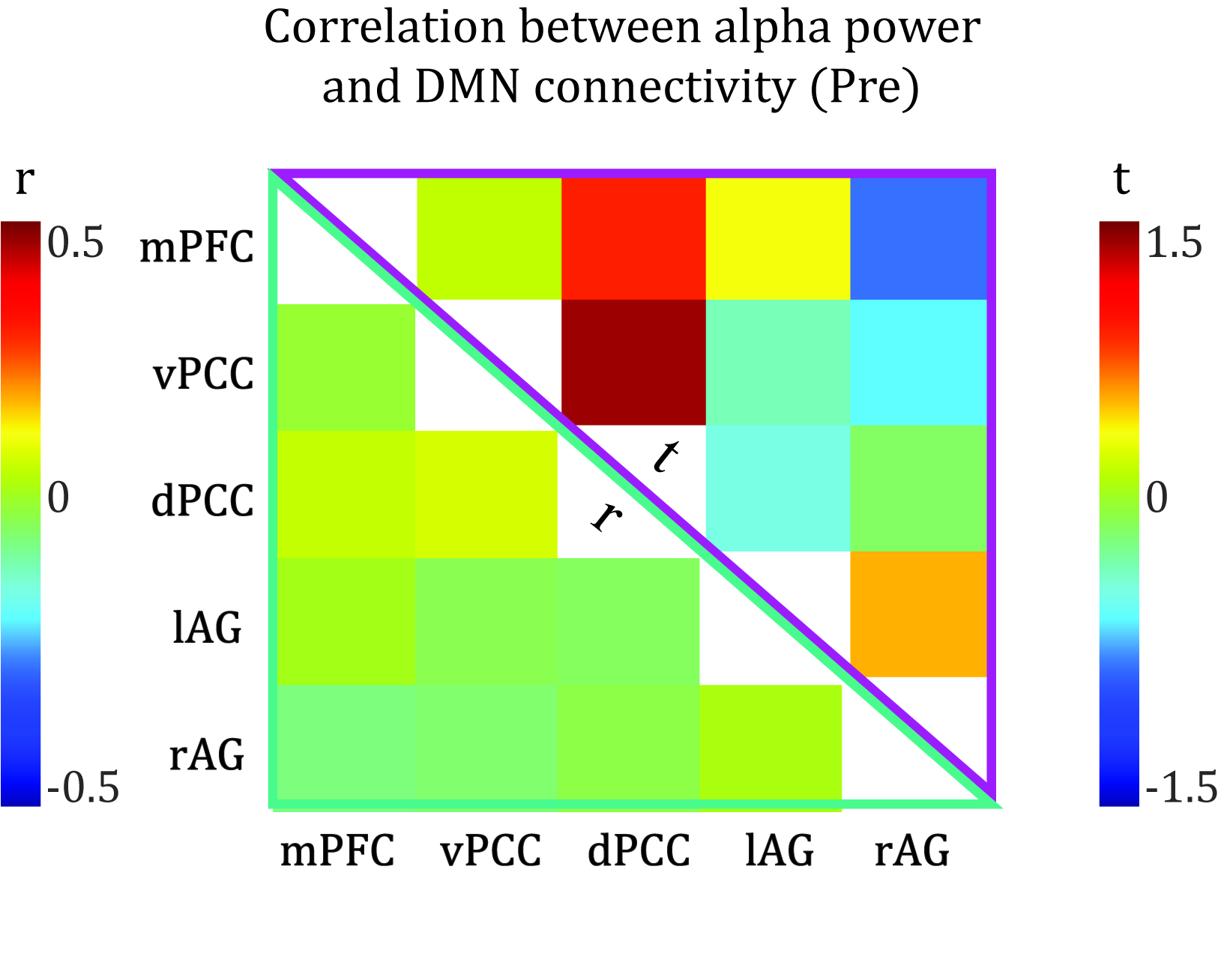

Supplement: Figure 2-2 — Baseline alpha-DMN Coupling: Baseline data pooled between the two groups showed no reliable coupling between alpha and DMN connectivity timeseries. The lower left and upper right portions of the matrix reflect the r and t values, respectively. Download Figure 2-2, TIF file. [file eneuro-12-ENEURO.0449-24.2025-s002.tif]
